# Author Correction: Developmental genetics of color pattern establishment in cats

**DOI:** 10.1038/s41467-021-25886-9

**Published:** 2021-09-16

**Authors:** Christopher B. Kaelin, Kelly A. McGowan, Gregory S. Barsh

**Affiliations:** 1 HudsonAlpha Institute for Biotechnology, Huntsville, AL USA; 2 Department of Genetics, Stanford University School of Medicine, Stanford, CA USA

**Keywords:** RNA sequencing, Cat, Pattern formation, Development, Evolutionary biology

Correction to: *Nature Communications* 10.1038/s41467-021-25348-2, published online 7 September 2021.

The original version of this Article contained an error in the labels of <Fig. 5d>, which incorrectly read ‘<Dkk4^A18V^/Dkk4>’ and ‘<Dkk4^C63Y^/Dkk4>’. The correct version states ‘<Dkk4^A18V^>’ and ‘<Dkk4^C63Y^>’.

This has been corrected in both the PDF and HTML versions of the Article.

