# Supplementary material for: Developmental genetics of color pattern establishment in cats
Source: Nat Commun. 2021 Sep 7;12:5127. doi: 10.1038/s41467-021-25348-2 (PMC8423757; doi:10.1038/s41467-021-25348-2)
Supplement: Supplementary file 1 — Supplementary Information [file 41467_2021_25348_MOESM1_ESM.pdf]

Supplementary Material for Kaelin, McGowan, and Barsh

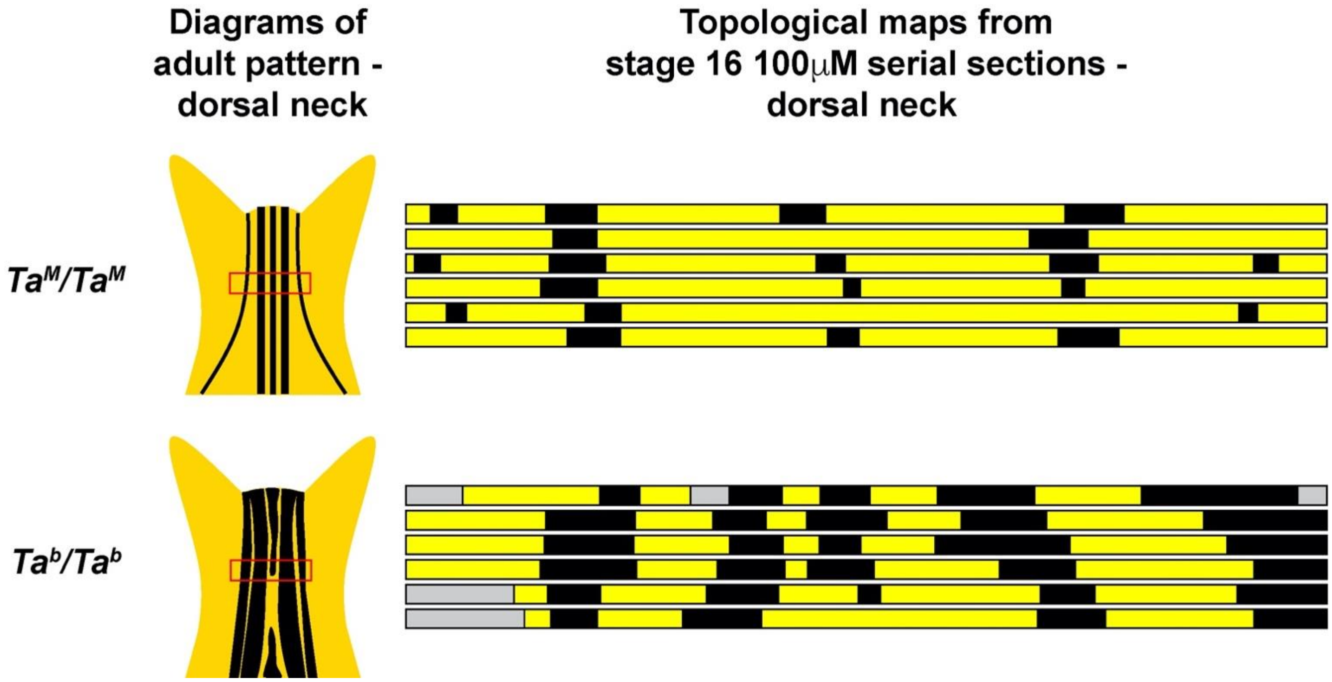

Supplementary Fig. 1. Topological maps of dorsal neck skin from Stage 16. Ta^M/Ta^M and Ta^b/Ta^b embryos (thin epidermis, yellow; thick epidermis, black; no data, grey). Each map represents six serial sections from an embryo of the indicated genotype; thick and thin areas in each section were marked independently and then aligned to generate a two-dimensional representation of the topology. Cartoons (left) show adult pigmentation pattern on the dorsal neck (red box indicates anatomic location of map).

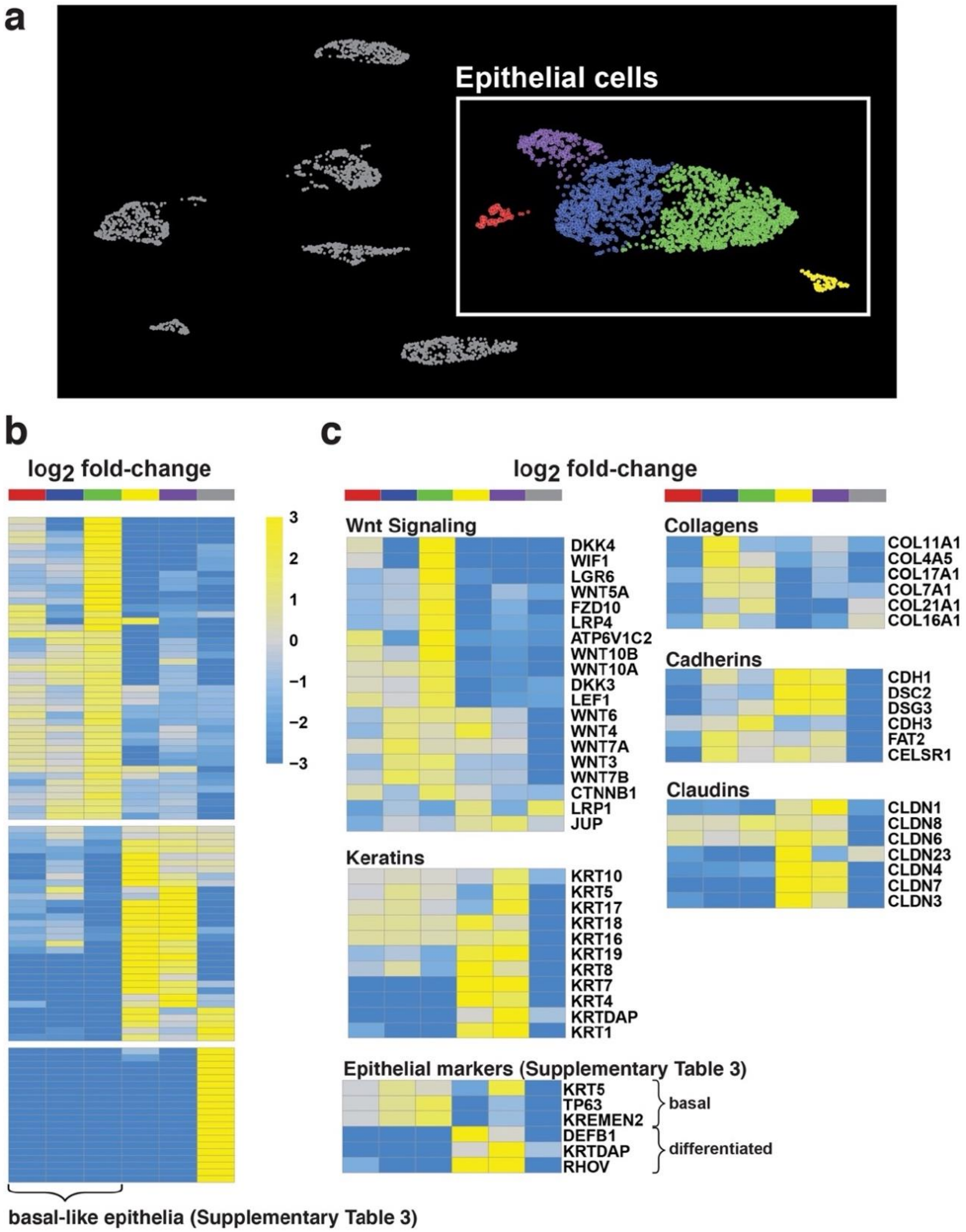

Supplementary Figure 2. Patterns of gene expression in different cell types at stage 16a. (a) Cell populations defined by *k*-means clustering (at *k*=10) represent spatially distinct UMAP clusters (except for the basal keratinocyte subpopulations, whose defining characteristics are described in the text). **(b)** To characterize the different epithelial cell populations, the top 20 upregulated genes in each population were selected, and the $\log_2$-fold change in expression relative to the mean expression across all cells was used to cluster the union of these genes (n=97) and cell populations using the pheatmap (v1.012) R package and Ward's method. Non-epithelial cell populations are included in all analyses but are

considered as a single group, indicated in grey. This approach identifies three major population groups: basal-like epithelia (red, blue, green), non-basal epithelia (yellow, purple), and non-epithelial cells (grey), and corresponding sets of genes that define those groups. **(c)** Expression patterns in the different populations for individual genes in six different gene families. Differentially expressed genes were filtered for inclusion in heat maps by significance (FDR *q*-value < 0.05) and expression level (normalized mean expression > 0.25 transcripts/cell).

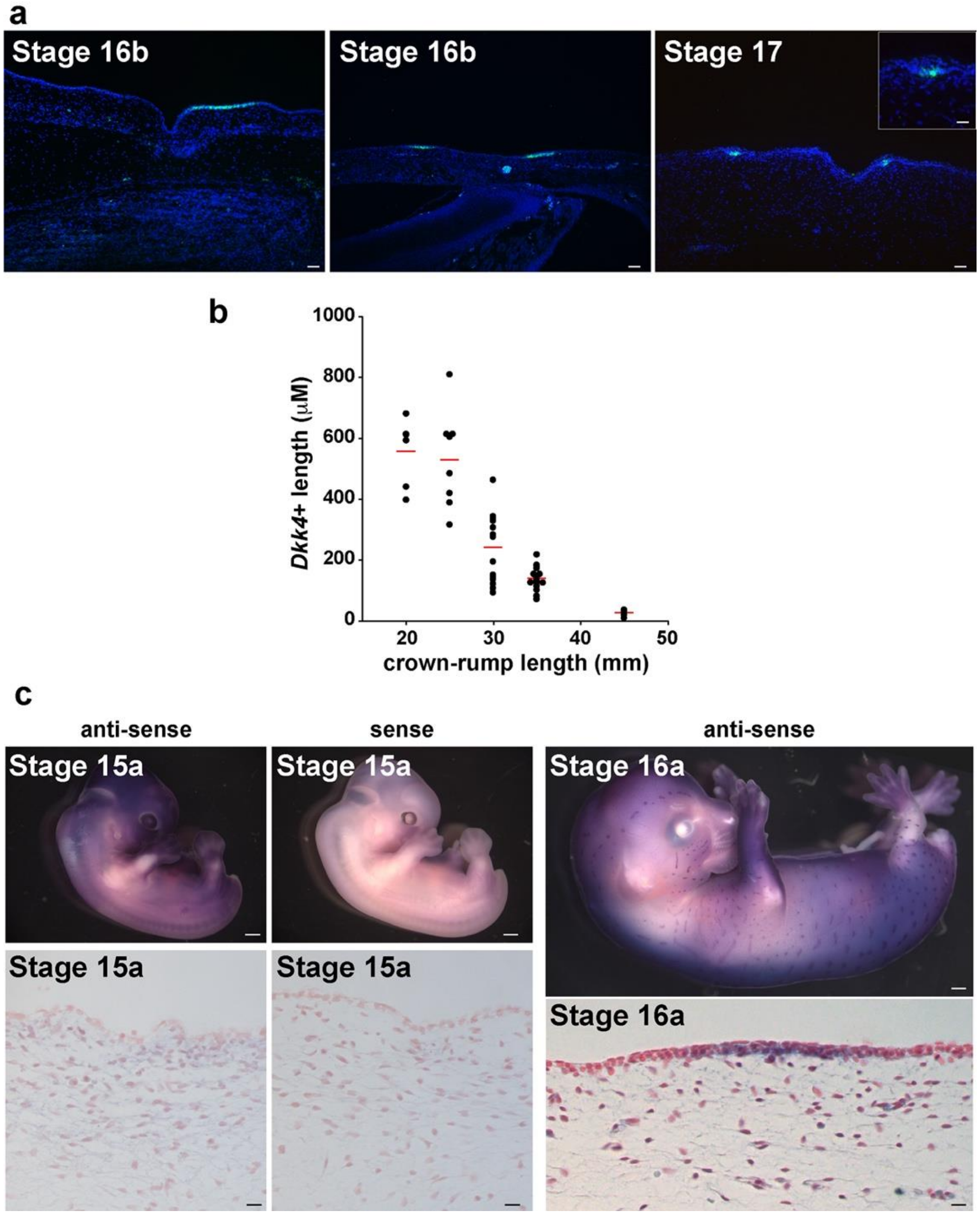

Supplementary Fig. 3. *Dkk4* expression in embryonic cat skin. (**a**) *Dkk4* expression (green) in sections of embryonic cat skin (DAPI, blue). 1-3 *Dkk4*-positive cells are found in sections of developing hair buds at stage 17 (right panel, inset). Observations were made independently on three or more Ta^{M}/- embryos from each developmental stage with similar results. (**b**) Length of *Dkk4*-positive regions from sections of embryonic skin at different developmental time points (crown-rump length is a surrogate for stage of embryonic development, Supplementary Table 1; length of 6-16 *Dkk4*-positive regions measured from at least three sections at each developmental time point, red bar denotes mean). (**c**) *Dkk4* expression (purple) in stage 15a and stage 16a embryos (top panels; sense control probe does not stain embryo). *In situ* hybridizations were carried out independently on four Ta^{M}/- embryos at each stage. Sections of skin (bottom panels) from embryos shown in top panels are stained with nuclear fast red. Scale bars: a, 50 μM; b inset, 25 μM; c top panels, 1mm; c bottom panels 25 μM.

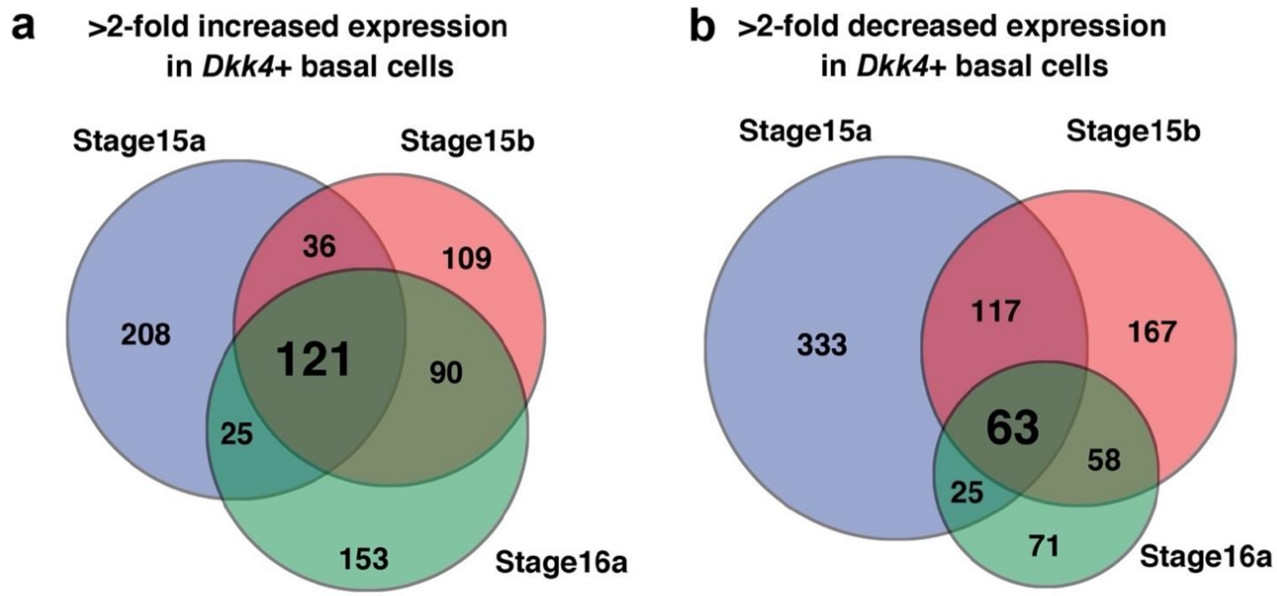

c

Gene	Fold-increase at stage:			Transcripts per cell:			Stage 16a q-value
	15a	15b	16a	15a	15b	16a	
Dkk4	504	436	17.7	22.3	99.2	35.6	6.8 e-42
Wif1	18.2	72.8	27.6	0.05	0.34	2.22	4.3 e-43
Dkk3	0.67	3.96	1.80	0.54	1.14	0.85	4.8 e-3
Atp6v1c2	9.42	31.6	8.34	0.31	1.51	1.32	1.4 e-21
Lgr6	3.13	5.93	3.89	0.79	0.83	0.84	1.2 e-8
Lrp4	3.22	3.15	3.92	2.41	3.03	2.56	8.2 e-10
Fzd10	3.63	2.58	3.29	2.58	4.12	1.86	2.2 e-7
Lef1	3.31	2.03	2.21	3.09	4.82	2.83	0.0062
Wnt10b	1.80	1.51	3.27	9.06	9.88	3.11	1.8 e-7
Wnt5a	2.18	1.53	2.89	1.32	0.91	0.59	6.4 e-5
Ctnnb1	1.75	2.93	2.92	13.3	20.4	10.9	2.9 e-6

Supplementary Figure 4. Relationship between differentially expressed genes during fetal skin development highlights components of Wnt signaling. The gene list used for the analysis depicted here is more sensitive and less stringent than that depicted in Fig. 3 (Methods). Overlap of gene expression profiles for genes with >2-fold increased (**a**) or decreased (**b**) expression in *Dkk4*-positive basal keratinocytes at stages 15a (blue), 15b (red), and 16a (green). (**c**) Gene expression metrics for Wnt signaling genes with elevated expression in *Dkk4*-positive basal keratinocytes; q-values indicate the significance of differential expression between basal keratinocyte subpopulations at stage 16a (negative binomial exact test). Complete expression data is provided in Supplementary Data 1. Genes are colored according to their predicted role in either short-range activation (blue) or long-range inhibition (red) of Wnt signaling in thick and thin epidermis, respectively, as presented in a model for color pattern establishment in Fig. 3d.

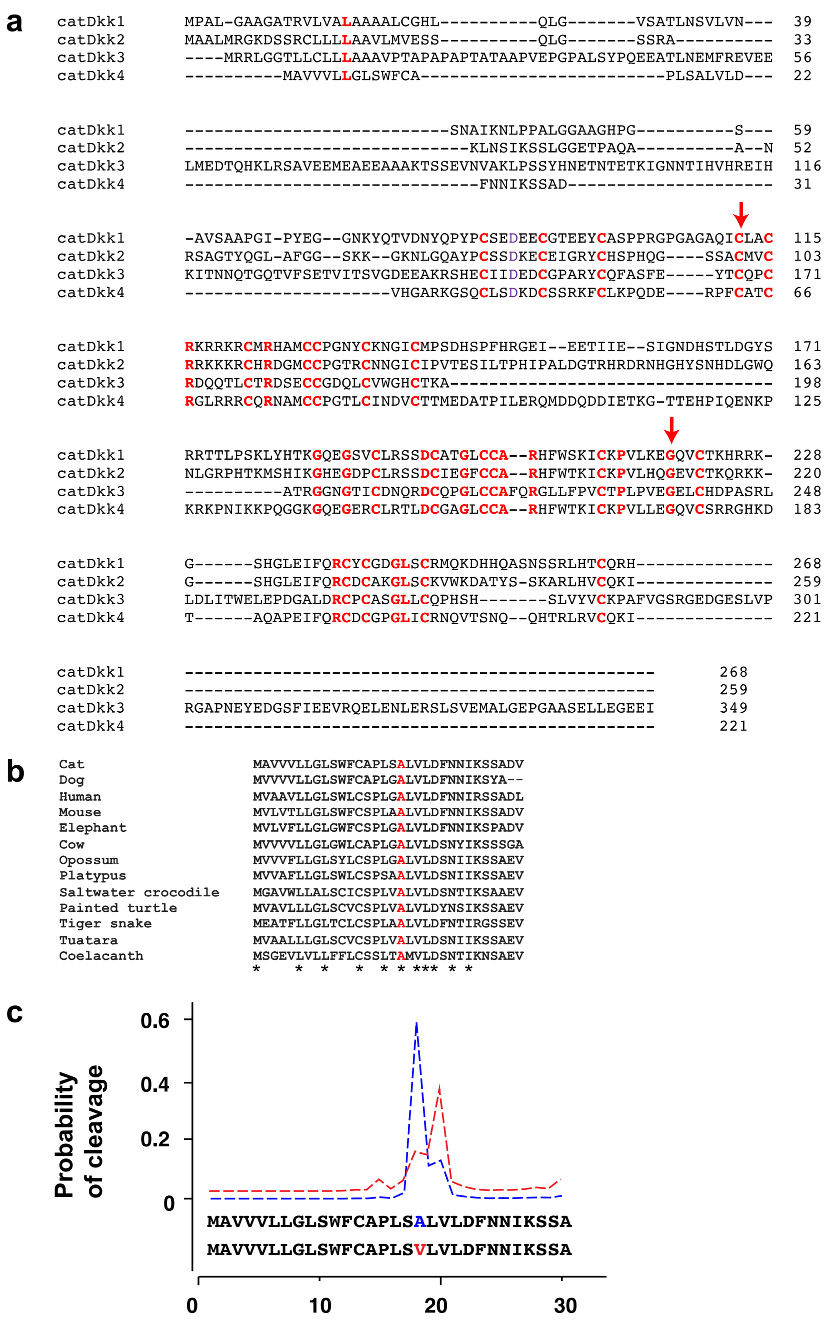

Supplementary Fig. 5. Evolutionary constraint and functional prediction for cat Dkk4 variants associated with *Ticked*. (**a**) A protein alignment of cat Dkk paralogs, with conserved residues colored red, and arrows highlighting the position of one of the cat *Dkk4* mutations (p.Cys63Tyr) and the position of the potentially deleterious residue in tiger (p.Gly173Glu). **(b)** Vertebrate Dkk4 alignment with the residue that harbors the other cat *Dkk4* mutation (p.Ala18Val) colored red. **(c)** Signal peptide cleavage site efficiencies predicted by SignalP5.0 for p.Ala18 (blue) and p.Val18 (red) variants.

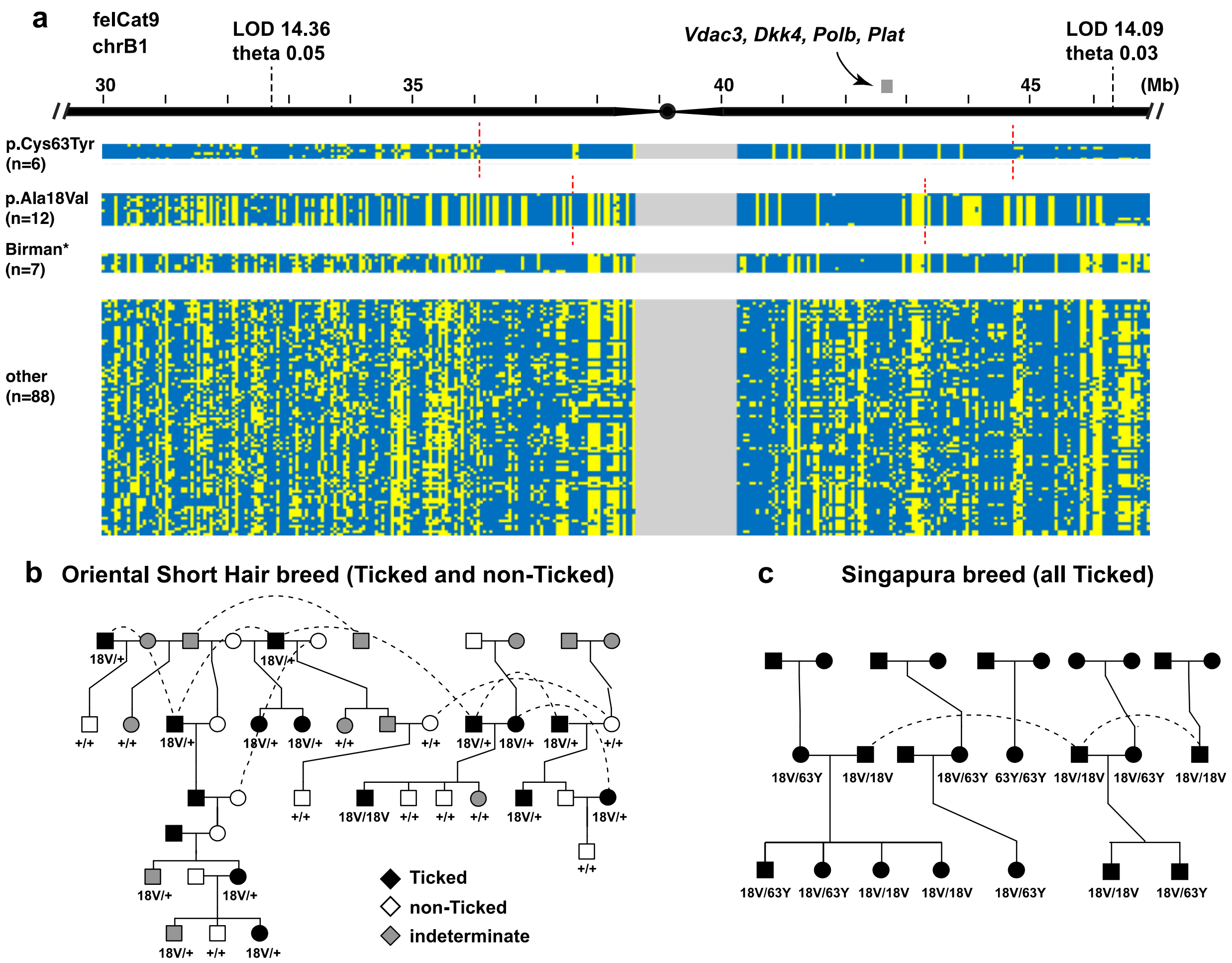

Supplementary Fig. 6. *Dkk4* variants occur on extended haplotypes and co-segregate with *Ticked* in pedigrees. (**a**) Haplotypes (rows) were inferred using Beagle (v4.1) for 57 cats in the 99 Lives data set, with SNPs (columns) indicated as reference (blue) or alternate (yellow) alleles. Broken black and red lines delineate the *Ticked* linkage interval and inferred ancestral recombination breakpoints on *Ticked* haplotypes, respectively. The grey bar indicates a region containing four genes that are differentially expressed between stage 16a basal keratinocyte subpopulations. The Birman* haplotype group consists of Birman (n=5), Devon Rex (n=1), and Domestic Short Hair (n=1) haplotypes, which are similar to the shared p.Ala18Val haplotype but do not carry the p.Ala18Val allele. **(b)** An Oriental Shorthair pedigree, demonstrating cosegregation of the Ticked pattern and the p.Ala18Val allele. **(c)** A Singapura pedigree, demonstrating fixation of the Ticked pattern as a consequence of p.Ala18Val and p.Cys63Tyr segregation.

```
Cheetah             MAVVFLLGLSWFCAPLSALVLDFNNIKSSANVHRAQKGSQCLSDKDCSSRKFCLKPQDER    60
Cougar              MAVVFLLGLSWFCAPLSALVLDFNNIKSSADVHGAQKGSQCLSDKDCSSRKFCLKPQDER    60
Tiger               MAVVVLLGLSWLCAPLSALVLDFNNIKSSADVQGARKGSQCLSDKDCSSRKFCLKPQDER    60
Fishing cat         MAVVVLLGLSWFCAPLSALVLDFNNIKSSADVHGARKGSQCLSDKDCSSRKFCLKPQEER    60
Jaguar              MAVVVLLGLSWFCAPLSALVLDFNNIKSSADVHGARKGSQCLSDKDCSSRKFCLKPQDER    60
Lion                MAVVVLLGLSWFCAPLSALVLDFNNIKSSADVHGARKGSQCLSDKDCSSRKFCLKPQDER    60
Snow leopard        MAVVVLLGLSWFCAPLSALVLDFNNIKSSADVHGARKGSQCLSDKDCSSRKFCLKPQDER    60
Leopard             MAVVVLLGLSWFCAPLSALVLDFNNIKSSADVHGARKGSQCLSDKDCSSRKFCLKPQDER    60
Asian leopard cat   MAVVVLLGLSWFCAPLSALVLDFNNIKSSADVHGARKGSQCLSDKDCSSRKFCLKPQDER    60
Clouded leopard     MAVVVLLGLSWFCAPLSALVLDFNNIKSSADVHGARKGSQCLSDKDCSSRKFCLKPQDER    60
Pallas cat          MAVVVLLGLSWFCAPLSALVLDFNNIKSSADVHGARKGSQCLSDKDCSSRKFCLKPQDER    60
Spanish lynx        MAVVVLLGLSWFCAPLSALVLDFNNIKSSADVHGARKGSQCLSDKDCSSRKFCLKPQDER    60
Pampas cat          MAVVVLLGLSWFCAPLSALVLDFNNIKSSADVHGARKGSQCLSDKDCSSRKFCLKPQDER    60
Canadian lynx       MAVVVLLGLSWFCAPLSALVLDFNNIKSSADVHGARKGSQCLSDKDCSSRKFCLKPQDER    60
Domestic cat        MAVVVLLGLSWFCAPLSALVLDFNNIKSSADVHGARKGSQCLSDKDCSSRKFCLKPQDER    60
Sand cat            MAVVVLLGLSWFCAPLSALVLDFNNIKSSADVHGARKGSQCLSDKDCSSRKFCLKPQDER    60
Jungle cat          MAVVVLLGLSWFCAPLSALVLDFNNIKSSADVHGARKGSQCLSDKDCSSRKFCLKPQDER    60
Black-footed cat    MAVVVLLGLSWFCAPLSALVLDFNNIKSSADVHGARKGSQCLSDKDCSSRKFCLKPQDER    60
                    ****.******:******************:*: *:*********************:**

Cheetah             PLCATCRGLRRRCQRNAMCCPGILCMNDVCTAMEDATPILERQMDDQDDIETKGTTEHPI    120
pumaDkk4            PFCATCRGLRRRCQRNAMCCPGTLCMNDVCTAMEDATPILERQMDDQDDIETKGTTEHPI    120
tigerDkk4           PFCATCRGLRRRCQRNAMCCPGTLCMNDVCTTMEDATPILERQMDDQDDIETKGTTEHPI    120
fishing_catDkk4     PFCATCRGLRRRCQRNAMCCPGTLCMNDVCTTMEDATPILERQMDDQDDIETKGTTEHPI    120
jaguarDkk4          PFCATCRGLQRRCQRNAMCCPGTLCMNDVCTTMEDATPILERQMDDQDDIETKGTTEHPI    120
lionDkk4            PFCATCRGLRRRCQRNAMCCPGTLCMNDVCTTMEDATPILERQMDDQDDIETKGTTEHPI    120
snow_leopardDkk4    PFCATCRGLRRRCQRNAMCCPGTLCMNDVCTTMEDATPILERQMDDQDDIETKGTTEHPI    120
leopardDkk4         PFCATCRGLRRRCQRNAMCCPGTLCMNDVCTTMEDATPILERQMDDQDDIETKGTTEHPI    120
alcDkk4             PFCATCRGLRRRCQRNAMCCPGTLCMNDVCTTMEDATPILERQMDDQDDIETKGTTEHPI    120
cloudedDkk4         PFCATCRGLRRRCQRNAMCCPGTLCMNDVCTTMEDATPILERQMDDQDDIETKGTTEHPI    120
pallas_catDkk4      PFCATCRGLRRRCQRNAMCCPGTLCMNDVCTTMEDATPILERQMDDQDDIETKGTTEHPI    120
SpanishLynxDkk4     PFCATCRGLRRRCQRNAMCCPGTLCMNDVCTTMEDATPILERQMDDQDDIETKGTTEHPI    120
pampasDkk4          PFCATCRGLRRRCQRNAMCCPGTLCMNDVCTTMEDATPILERQMDDQDDIETKGTTEHPI    120
CanadianLynxDkk4    PFCATCRGLRRRCQRNAMCCPGTLCMNDVCTTMEDATPILERQMDDQDDIETKGTTEHPI    120
catDkk4             PFCATCRGLRRRCQRNAMCCPGTLCINDVCTTMEDATPILERQMDDQDDIETKGTTEHPI    120
sand_catDkk4        PFCATCRGLRRRCQRNAMCCPGTLCINDVCTTMEDATPILERQMDDQDDIETKGTTEHPI    120
chausDkk4           PFCATCRGLRRRCQRNAMCCPGTLCINDVCTTMEDATPILERQMDDQDDIETKGTTEHPI    120
nigripesDkk4        PFCATCRGLRRRCQRNAMCCPGTLCINDVCTTMEDATPILERQMDDQDDIETKGTTEHPI    120
                    *:*******:************ **:*****:****************************

cheetahDkk4         QENKPKRKPNIKKPQDGKGQEGERCLRTLDCGAGLCCARHFWTKICKPVLLEGQVCSRRG    180
pumaDkk4            QENKPKRKPNIKKPQDGKGQEGERCLRTLDCGAGLCCARHFWTKICKPVLLEGQVCSRRG    180
tigerDkk4           QENKPKRKPNIKKPQGGKGQEGERCLRTLDCGAGLCCARHFWTKICKPVLLEEQVCSRRG    180
fishing_catDkk4     QENKPKRKPNIKKPQGGKGQEGERCLRTLDCGAGLCCARHFWTKICKPVLLEGQVCSRRG    180
jaguarDkk4          QENKPKRKPNIKKPQGGKGQEGERCLRTLDCGAGLCCARHFWTKICKPVLLEGQVCSRRG    180
lionDkk4            QENKPKRKPNIKKPQGGKGQEGERCLRTLDCGAGLCCARHFWTKICKPVLLEGQVCSRRG    180
snow_leopardDkk4    QENKPKRKPNIKKPQGGKGQEGERCLRTLDCGAGLCCARHFWTKICKPVLLEGQVCSRRG    180
leopardDkk4         QENKPKRKPNIKKPQGGKGQEGERCLRTLDCGAGLCCARHFWTKICKPVLLEGQVCSRRG    180
alcDkk4             QENKPKRKPNIKKPQGGKGQEGERCLRTLDCGAGLCCARHFWTKICKPVLLEGQVCSRRG    180
cloudedDkk4         QENKPKRKPNIKKPQGGKGQEGERCLRTLDCGAGLCCARHFWTKICKPVLLEGQVCSRRG    180
pallas_catDkk4      QENKPKRKPNIKKPQGGKGQEGERCLRTLDCGAGLCCARHFWTKICKPVLLEGQVCSRRG    180
SpanishLynxDkk4     QENKPKRKPNIKKPQGGKGQEGERCLRTLDCGAGLCCARHFWTKICKPVLLEGQVCSRRG    180
pampasDkk4          QENKPKRKPNIKKPQGGKGQEGERCLRTLDCGAGLCCARHFWTKICKPVLLEGQVCSRRG    180
CanadianLynxDkk4    QENKPKRKPNIKKPQGGKGQEGERCLRTLDCGAGLCCARHFWTKICKPVLLEGQVCSRRG    180
catDkk4             QENKPKRKPNIKKPQGGKGQEGERCLRTLDCGAGLCCARHFWTKICKPVLLEGQVCSRRG    180
sand_catDkk4        QENKPKRKPNIKKPQGGKGQEGERCLRTLDCGAGLCCARHFWTKICKPVLLEGQVCSRRG    180
chausDkk4           QENKPKRKPNIKKPQGGKGQEGERCLRTLDCGAGLCCARHFWTKICKPVLLEGQVCSRRG    180
nigripesDkk4        QENKPKRKPNIKKPQGGKGQEGERCLRTLDCGAGLCCARHFWTKICKPVLLEGQVCSRRG    180
                    ***************.************************************ *******

cheetahDkk4         HKDTAQAPEIFQRCDCGPGLICRNQVTGNQQHTRLRVCQKI--  221
pumaDkk4            HKDTAQAPEIFQRCDCGPGLICRNQVTSNQQHTRLRVCQKI--  221
tigerDkk4           HKDTAQAPEIFQRCDCGPGLICRNQVTGNQQHTRLRVCQKI--  221
fishing_catDkk4     HKDTAQAPEIFQRCDCGPGLICRNQVTGNQQHTRLRVCQKI--  221
jaguarDkk4          HKDTAQAPEIFQRCDCGPGLICRNQVTGNQQHTRLRVCQKI--  221
lionDkk4            HKDTAQAPEIFQRCDCGPGLICRNQVTGNQQHTRLRVCQKNLN  223
snow_leopardDkk4    HKDTAQAPEIFQRCDCGPGLICRNQVTGNQQHTRLRVCQKI--  221
leopardDkk4         HKDTAQAPEIFQRCDCGPGLICRNQVTGNQQHTRLRVCQKI--  221
alcDkk4             HKDTAQAPEIFQRCDCGPGLICRNQVTGNQQHTRLRVCQKI--  221
cloudedDkk4         HKDTAQAPEIFQRCDCGPGLICRNQVTGNQQHTRLRVCQKI--  221
pallas_catDkk4      HKDTAQAPEIFQRCDCGPGLICRNQVTGNQQHTRLRVCQKI--  221
SpanishLynxDkk4     HKDTAQAPEIFQRCDCGPGLICRNQVTGNQQHTRLRVCQKI--  221
pampasDkk4          HKDTAQAPEIFQRCDCGPGLICRNQVTGNQQHTRLRVCQKI--  221
CanadianLynxDkk4    HKDTAQAPEIFQRCDCGPGLICRNQVTSNQQHTRLRVCQKI--  221
catDkk4             HKDTAQAPEIFQRCDCGPGLICRNQVTSNQQHTRLRVCQKI--  221
sand_catDkk4        HKDTAQAPEIFQRCDCGPGLICRNQVTSNQQHTRLRVCQKI--  221
chausDkk4           HKDTAQAPEIFQRCDCGPGLICRNQVTGNQQHTRLRVCQKI--  221
nigripesDkk4        HKDTAQAPEIFQRCDCGPGLICRNQVTGNQQHTRLRVCQKI--  221
                    ***************************.************
```

Supplementary Fig. 7. Protein alignment of predicted Dkk4 protein sequence from 29 felid species. A summary of derived variants and predicted deleteriousness is given in Supplementary Table 9.

Supplementary Table 1. Stages of embryonic and fetal development in the domestic cat.

Prenatal stage (cat)[a, b]	Days post-coitus[a]	Crown-rump length (mm)[b]	Prenatal stage (mouse)[c]	Anatomic features (cat)[a,b]	Histologic features (cat)[b]	Molecular features (cat)[b]
13	19-21	8-11	E11		monolayer of epidermal cells	
14	21-23	11-15	E12			
15a	23-25[a,b]	15-17	E13	finger web resorption		
15b	23-25[a,b]	18-21	E14	toe web resorption		*Dkk4*-positive domains in epidermis
16a	25-28[a,b]	22-26	E15	eye lid closure begins	“thick” epidermal domains	*Dkk4*-positive “thick” domains
16b	25-28[a,b]	27-37	E15			Krt10-positive cells in “thick” domains
17	28-32	38-47	E16	eyelid closure complete	hair follicle buds and pegs	*Dkk4*-positive cells in hair buds
18	32-38	48-60			epidermis uniform thickness	Krt10 uniformly expressed in epidermis
19	38-44	61-80	E17			
20	44-48	81-90				
21	48-60	91-109		hair eruption; pigment pattern visible	pigmented hair bulbs	
22	60-66	110-130	P4-6			

[a] Based on Knospe et al. [1]
[b] Based on data reported here
[c] Based on Kaufman et al. [2]

Supplementary Table 2. Histologic and molecular features of thick epidermal domains in cat as compared to developing hair follicle placodes and "normal" epidermal stratification.

	"Thick" epidermal domain (cat[a])	Hair follicle placode (mouse, cat[a])	Developing epidermis (mouse, cat[a])
**Tissue organization**	3-5 layers of uniformly spaced, basaloid cells	Single layer of crowded, basal cells below second layer of basal-like cells, intermediate cell layer and periderm [3]*	Single layer of uniformly spaced basal cells below intermediate cell layer and periderm [4]; intermediate cell layer differentiates into spinous, granular, cornified layers; periderm sheds [4]
**Cellular morphology**	Round, basaloid cells uniformly spaced	Vertically polarized, basal cells undergo compaction [3,5]; round, basaloid and intermediate cells	Round, basal and intermediate cells uniformly spaced [4]; differentiated epidermal cells flatten
**Domain width**	50-500 µM (~20-200 cells); $Ta^b/Ta^b > Ta^M/-$	~50 µM (~5-20 cells) [3]	
**Domain depth**	No invagination into dermis	Invaginates into dermis [3]*	
**Proliferation**	Basal and supra-basal cells proliferate (31% Ki67-positive)	Small fraction proliferating cells (~8%, thickening due to cell migration) [5]; basal cells proliferate; supra-basal cells proliferate during invagination [5,6]	Basal and few supra-basal cells located just above the basal cell layer proliferate [4,7]
**Keratin expression pattern**	Krt5-positive basal and supra-basal cells; Krt10-positive cells lie above 3-5 layers of Krt5-positive cells	Krt5-positive basal and basaloid cells; Krt10-positive intermediate cell layer; Krt8/18-positive periderm [4]	Krt5-positive basal cells [4]; Krt1/10-positive supra-basal cells [4,7]; Krt8/18-positive periderm [4]
**Dermal cell arrangement**	Dermal cells evenly distributed	Dermal cells cluster below placode [3]	Dermal cells evenly distributed

[a]Data in cat from this study.

Supplementary Table 3. Gene markers used for identification of UMAP clusters.

Cell population	Key genes	Reference
Macrophage/dendritic cells	*Cd163*, *C1qa*, *Cd14*	[8,9]
Neural Crest	*Fabp7*, *Sox10*, *Foxd3*	[10]
Stratified epithelium/periderm	*Krtdap*, *Rhov*, *Defb1*	[11]
Basal keratinocytes	*Krt5*, *Tp63*, *Kremen2*	[12]
Dermal fibroblasts	*Lum*, *Twist1*, *Twist2*	[12]
Myoblasts	*Myf5*, *Myod1*, *Msc*	[13,14]
Endothelium	*Pecam1*, *Cdh5*, *Tie1*	[15]
Vascular endothelium	*Cd34*, *Esam*, *Fam198b*	[16]
Lymphatic endothelium	Ccl21, Mmrn1, Igf1	[17]

Supplementary Table 4. scRNAseq summary statistics.

	Embryonic stage		
	15a	15b	16a
ID	C16	C14	C64
10x Genomics Chemistry	v3	v3	v2
Total reads	455,308,860	450,083,295	404,331,199
% mapped to genome	87.9	87.3	87.9
% mapped to transcriptome	58.9	60.1	63.5
Estimated cell count	5,493	4,454	4,617
Mean reads per cell	82,889	101,051	87,574
Median genes per cell	4,119	4,180	2,570
Median UMI per cell	18,244	17,966	9,419
Total genes detected	25,514	25,222	24,164
Estimated cell number	5,493	4,454	4,617
Keratinocyte cell number	1,791	2,425	2,515
Keratinocyte cell fraction	0.33	0.54	0.54

Supplementary Table 5. *Dkk4* coding variants detected in 57 cats[a].

felCat9 coordinate	cDNA variant[b]	Amino acid variant[b]	Allele freq (n=57)[c]	Abyssinian allele freq (n=4)[c]	CADD phred
chrB1:42620835	c41t	A18V	0.10	0.13	22.9
chrB1:42621481	g188a	C63Y	0.05	0.75	21.4
chrB1:42621505	g212a	R71K	0.14	0.75	5.8
chrB1:42621551	a258g	I86M	0.01	0	0.3[d]
chrB1:42622162	a395g	K132R	0.12	0	14.2
chrB1:42623424	t602c	I201T	0.66	0.13	16.0[e]
chrB1:42623444	a622g	S208G	0.01	0	0.2
chrB1:42623455	a633c	Q211H	0.01	0	0.2[d]

[a] All variants predicted to alter amino acids in Dkk4 are shown. In 57 cat genome sequences from the 99 Lives collection, there are an additional 3 variants in *Dkk4* that are synonymous, and with CADD scores of Q<10. No variants predicted to alter amino acids were identified in Abyssinians for the 3 other genes that are differentially expressed and overlap with the *Ticked* linkage interval, *Plat*, *Polb*, and *Vdac3*.

[b] Ancestral allele is shown as the reference allele.

[c] Allele frequencies for the derived allele.

[d] Reference base between cat and human differs.

[e] Adjacent nucleotide position differs between cat and human, affecting the reference alternate codon, making CADD score interpretation unreliable.

Supplementary Table 6. *Dkk4* alleles categorized according to breed and phenotype[a].

	Dkk4 genotype						
Breed and phenotype	+/+	+/18V	+/63Y	18V/18V	18V/63Y	63Y/63Y	Total
Abyssinian (Ticked)	0	0	1	0	2	34	37
Singapura (Ticked)	0	1	1	4	16	4	26
Burmese (Ticked)	1	1	0	11	0	0	13
Mau (non-Ticked)	8	0	0	0	0	0	8
Ocicat (non-Ticked)	13	0	0	0	0	0	13
Bengal (non-Ticked)	10[b]	0	0	0	0	0	10[b]
OSH and OLH Ticked	0	23	0	2	0	0	25
OSH and OLH non-Ticked	21	0	0	0	0	0	21
Non-breed cats Ticked[c]	0	4	0	0	0	0	4
Non-breed cats non-Ticked[c]	182	0	0	0	0	0	182

[a] The p.Ala18Val and p.Cys63Tyr variants are indicated as 18V and 63Y, respectively. The table includes data from 18 breed cats in the 99Lives dataset (4 Abyssinian, 3 Egyptian Mau, 1 Ocicat, 5 Bengal, 5 Burmese), for which photographs were not available, and for which phenotype was inferred based on breed identity; all other data is based on samples that we ascertained and collected. Of the Oriental Shorthair (OSH) and Oriental Longhair (OLH), 15 had an indeterminate phenotype for which we could not infer *Ticked* genotype, and one was excluded due to an inconsistency in Mendelian transmission.

[b] Data shown are from targeted genotyping or high-coverage WGS. In additional data from low-coverage (0.1x – 0.5x) WGS on 526 Bengal cats, neither *Dkk4* variant was observed.

[c] Variation in other coat color genes that masks tabby pattern (as in a Siamese or non-agouti cat) can make it difficult to assess Ticked, and 6 non-breed cats were excluded from this table because their tabby pattern (or lack thereof) could not be determined.

Supplementary Table 7. *Dkk4* variants and predicted impact (CADD) in the Felidae.

Position[a]	Ref	Alt	Amino acid	Species	CADD
42377041	A	G	p.A2V	Tigrina	3.393
42377033	C	A	p.V5F	Puma, Jaguarundi, Cheetah	0.736
42376989	C	G	p.D58E	Fishing cat	14.5
42377012	G	A	p.F12L	Tiger	10.88
42376955	C	T	p.D31N	Cheetah	14.02
42376947	A	G	p.H32Q	Tiger	0.68
42376946	C	T	p.G33R	Cheetah	10.84
42376939	C	T	p.R35Q	Puma, Jaguarundi, Cheetah	9.835
42375756	G	T	p.F62L	Cheetah	11.17
42375733	C	T	p.R70Q	Jaguar	1.566
42375694	G	A	p.T83I	Cheetah	8.476
42374918	G	T	p.M86I	Domestic cat, Sand cat, Black-footed cat, Jungle cat	4.527
42374902	T	C	p.T92A	Puma, Jaguarundi, Cheetah, Bay cat, Asian gold., Marbled cat	0.571
42374889	G	C	p.A96G	Jaguarundi	4.66
42374887	T	C	p.T97A	Serval	0.771
42374845	G	T	p.E111K	Flat-headed cat	0.108
42374769	C	T	p.G136D	Puma, Jaguarundi, Cheetah	3.221
42374318	G	C	p.A153S	Flat-headed cat	10.67
42374257	C	T	p.G173E	Tiger	26.9
42374174	G	T	p.I201V	Geoffroys cat, Tigrina	2.552
42374153	T	C	p.G208S	Domestic cat, Sand cat, Puma	0.21
42374114	T	C	p.I221V	Bay cat	0.435
42374114	T	TT	p.I221fsX4	Lion	19.07

[a] Genomic coordinates from chromosome B1, felCat9 assembly

Supplementary Table 8. Oligonucleotide primers for genotyping *Taqpep* and *Dkk4* variation.

Variant	Primer
Taqpep p.Trp841X	GCCTTCGGAAGTGATGAAGA
	ACTTCAGATTCCGCCACAAC
Dkk4 p.Ala18Val	GAGCTGAGAAGGTCAAGGTGA
	GTGGGTACTTGTGCCATTCC
Dkk4 p.Cys63Try	CCACTGTGATTTGGCTTCCT
	CAGTCCCACAGGGGTTTATG

Supplementary Table 9. Basal keratinocyte subpopulation cell number at different *Dkk4* expression thresholds.

Stage	Subpopulation	Expression Threshold			
		2-fold	4-fold	8-fold	16-fold
15a	*Dkk4*-	1641	1569	1670	1682
15a	*Dkk4*+	77	59	48	36
15b	*Dkk4*-	824	865	916	980
15b	*Dkk4*+	758	717	666	602
16a	*Dkk4*-	846	931	1018	1193
16a	*Dkk4*+	1216	1131	1044	869

1. Knospe, C. Periods and Stages of the Prenatal Development of the Domestic Cat. *Anatomia, Histologia, Embryologia: Journal of Veterinary Medicine Series C* **31**, 37-51 (2002).
2. Kaufman, M. H. *The Atlas of Mouse Development* (Academic Press, 1992).
3. Paus, R. et al. A comprehensive guide for the recognition and classification of distinct stages of hair follicle morphogenesis. *J Invest Dermatol* **113**, 523-532 (1999).
4. Koster, M. I. & Roop, D. R. Mechanisms regulating epithelial stratification. *Annu Rev Cell Dev Biol* **23**, 93-113 (2007).
5. Ahtiainen, L. et al. Directional cell migration, but not proliferation, drives hair placode morphogenesis. *Dev Cell* **28**, 588-602 (2014).
6. Schmidt-Ullrich, R. et al. NF-kappaB transmits Eda A1/EdaR signalling to activate Shh and cyclin D1 expression, and controls post-initiation hair placode down growth. *Development* **133**, 1045-1057 (2006).
7. Lechler, T. & Fuchs, E. Asymmetric cell divisions promote stratification and differentiation of mammalian skin. *Nature* **437**, 275-280 (2005).
8. Murray, P. J. & Wynn, T. A. Protective and pathogenic functions of macrophage subsets. *Nat Rev Immunol* **11**, 723-737 (2011).
9. Shih, B. B. et al. Derivation of marker gene signatures from human skin and their use in the interpretation of the transcriptional changes associated with dermatological disorders. *J Pathol* **241**, 600-613 (2017).
10. Simões-Costa, M., Stone, M. & Bronner, M. E. Axud1 Integrates Wnt Signaling and Transcriptional Inputs to Drive Neural Crest Formation. *Dev Cell* **34**, 544-554 (2015).
11. Joost, S. et al. Single-Cell Transcriptomics Reveals that Differentiation and Spatial Signatures Shape Epidermal and Hair Follicle Heterogeneity. *Cell Syst* **3**, 221-237.e9 (2016).
12. Sennett, R. et al. An Integrated Transcriptome Atlas of Embryonic Hair Follicle Progenitors, Their Niche, and the Developing Skin. *Dev Cell* **34**, 577-591 (2015).
13. Chal, J. & Pourquié, O. Making muscle: skeletal myogenesis in vivo and in vitro. *Development* **144**, 2104-2122 (2017).
14. Fanzani, A. et al. Hypertrophy and atrophy inversely regulate Caveolin-3 expression in myoblasts. *Biochem Biophys Res Commun* **357**, 314-318 (2007).
15. Korhonen, E. A. et al. Tie1 controls angiopoietin function in vascular remodeling and inflammation. *J Clin Invest* **126**, 3495-3510 (2016).
16. Fina, L. et al. Expression of the CD34 gene in vascular endothelial cells. *Blood* **75**, 2417-2426 (1990).
17. Feng, W., Chen, L., Nguyen, P. K., Wu, S. M. & Li, G. Single Cell Analysis of Endothelial Cells Identified Organ-Specific Molecular Signatures and Heart-Specific Cell Populations and Molecular Features. *Front Cardiovasc Med* **6**, 165 (2019).
