# Supplementary material for: Developmental genetics of color pattern establishment in cats
Source: Nat Commun. 2021 Sep 7;12:5127. doi: 10.1038/s41467-021-25348-2 (PMC8423757; doi:10.1038/s41467-021-25348-2)
Supplement: Supplementary file 3 — Description of Additional Supplementary Files [file 41467_2021_25348_MOESM3_ESM.docx]

**Legend for Supplementary Data 1.** The Excel workbook consists of:

Worksheets 1,2, and 3: genes analyzed in the single-cell RNA-seq experiments at stages 15a, 15b, and 16a, analyzed for differential expression between *Dkk4*-positive and *Dkk4*-negative basal keratinocytes as described in the text.

Worksheet 4: list of 121 genes upregulated in *Dkk4*-positive basal keratinocytes as described in the text and Supplementary Fig. 4a and that serve as a signature for color pattern establishment, with log_2_ fold change values at stage 16a, compared to log_2_ fold change values for the same genes as previously determined during hair follicle placode development (Tomann et al., 2018, ref 30).

Worksheets 5, 6, and 7: lists of genes and associated expression data that correspond to clusters A, B, and C, respectively, from Fig. 3a.

Worksheets 8 and 9: lists of genes and associated expression data that correspond to Supplementary Fig. 4A and 4B, respectively.

Expression data provided in the worksheets include mean transcripts per cell, log_2_ fold change, nominal two-sided p-value and false discovery rate (q-value), based on a negative binomial exact test implemented in the Cell Ranger software (10x Genomics) as described in the text.
