# Supplementary material for: Developmental genetics of color pattern establishment in cats
Source: Nat Commun. 2021 Sep 7;12:5127. doi: 10.1038/s41467-021-25348-2 (PMC8423757; doi:10.1038/s41467-021-25348-2)
Supplement: Supplementary file 5 — Reporting Summary [file 41467_2021_25348_MOESM5_ESM.pdf]

nature research

Corresponding author(s): Greg Barsh

Last updated by author(s): May 28, 2021

Reporting Summary

Nature Research wishes to improve the reproducibility of the work that we publish. This form provides structure for consistency and transparency in reporting. For further information on Nature Research policies, see our Editorial Policies and the Editorial Policy Checklist.

Statistics

For all statistical analyses, confirm that the following items are present in the figure legend, table legend, main text, or Methods section.

n/a	Confirmed	
☐	☒	The exact sample size (*n*) for each experimental group/condition, given as a discrete number and unit of measurement
☐	☒	A statement on whether measurements were taken from distinct samples or whether the same sample was measured repeatedly
☐	☒	The statistical test(s) used AND whether they are one- or two-sided<br>*Only common tests should be described solely by name; describe more complex techniques in the Methods section.*
☒	☐	A description of all covariates tested
☐	☒	A description of any assumptions or corrections, such as tests of normality and adjustment for multiple comparisons
☐	☒	A full description of the statistical parameters including central tendency (e.g. means) or other basic estimates (e.g. regression coefficient) AND variation (e.g. standard deviation) or associated estimates of uncertainty (e.g. confidence intervals)
☐	☒	For null hypothesis testing, the test statistic (e.g. *F*, *t*, *r*) with confidence intervals, effect sizes, degrees of freedom and *P* value noted<br>*Give P values as exact values whenever suitable.*
☒	☐	For Bayesian analysis, information on the choice of priors and Markov chain Monte Carlo settings
☐	☒	For hierarchical and complex designs, identification of the appropriate level for tests and full reporting of outcomes
☒	☐	Estimates of effect sizes (e.g. Cohen's *d*, Pearson's *r*), indicating how they were calculated

Our web collection on statistics for biologists contains articles on many of the points above.

Software and code

Policy information about availability of computer code

Data collection	Leica DMRXA2 inverted microscope, Leica DFC550 digital camera, Leica LASV4 software package for microscopy images v4.2.0; SpectraMax iD3 plate reader and BioRad ChemiDoc Imaging System for Western blot quantitation); Illumina HiSeqX software.
Data analysis	BioRad Image Lab Software Suite v6.1; 10x Genomics Single Cell Software Suite (Cell Ranger and Loupe Browser, v3.1); R v3.6.1-4.0.3; Enrichr; Platypus, v0.1.5; SnpEff & SnpSift, v4.3i; CADD, v1.4; SignalP-5.0; BEAGLE, v.4.1; VCFtools, v0.1.16; PyMol browser v2.3.3; pheatmap (v1.012) R package, BWA (v0.7.16), SignalP-5.0

For manuscripts utilizing custom algorithms or software that are central to the research but not yet described in published literature, software must be made available to editors and reviewers. We strongly encourage code deposition in a community repository (e.g. GitHub). See the Nature Research guidelines for submitting code & software for further information.

Data

Policy information about availability of data

All manuscripts must include a data availability statement. This statement should provide the following information, where applicable:

- Accession codes, unique identifiers, or web links for publicly available datasets
- A list of figures that have associated raw data
- A description of any restrictions on data availability

Structure data for the N-terminal region of Dkk4 used for visualization is available at the Protein Data Bank (accession 5O57. For single-cell RNAseq, raw and processed data sets generated in this study have been deposited in the Gene Expression Omnibus database have been deposited in GEO (accession number: GSE152946). GEO files include unfiltered feature-barcode matrices in HDF5 format, output by the CellRanger pipeline, and Illumina fastq files for stage 15a, 15b, and 16a single-cell RNAseq.

April 2020

Field-specific reporting

Please select the one below that is the best fit for your research. If you are not sure, read the appropriate sections before making your selection.

- [x] Life sciences
- [ ] Behavioural & social sciences
- [ ] Ecological, evolutionary & environmental sciences

For a reference copy of the document with all sections, see nature.com/documents/nr-reporting-summary-flat.pdf

Life sciences study design

All studies must disclose on these points even when the disclosure is negative.

Sample size	For histologic, immunostaining, and in situ hybridization experiments on fetal cat tissues, sample sizes were chosen based on availability of field samples and experimental design, with a goal of minimizing type I error. Sample sizes for all experiments are indicated in the text and in Source Data, and consisted of at least 3 independent biologic samples except where only one or two samples, e.g. a rare genotype, was available. In the majority of experiments for which 3 or more independent samples were available, affirmative conclusions are based on the statistical significance of observed differences; no conclusions were reached based on acceptance of a null hypothesis (type II error). In a minority of experiments with sample size < 3, qualitative observations are reported (FIg. 2e, 6b), and are consistent with quantitative conclusions based on alternative approaches (Fig. 2d). For single cell RNA-sequencing, >4000 cells were capture from a single embryo at each of three developmental stages (15a, 15b, 16a). For genetic studies, 105 and 234 Ticked and nonTicked samples were collected and genotyped for Dkk4 variants.
Data exclusions	For single cell RNA-sequencing at stage 15b, a subset of basal keratinocytes had a distinct gene expression signature characterized by the expression of Engrailed (En1) and HoxC genes, likely to reflect cells from the developing limb bud. The En1/HoxC signature was not observed at stages 15a or 16a, and therefore this population of cells was excluded from the stage15b differential expression analysis.
Replication	All attempts at replication were successful. The exact number of times each experiment was performed is stated in the corresponding figure legend or in the methods.
Randomization	This is not a prospective study with an intervention so randomization is not applicable.
Blinding	Single expert individuals carried out the embryonic (KAM) and genomic (CBK) analyses and blinding would not have been appropriate or feasible.

Reporting for specific materials, systems and methods

We require information from authors about some types of materials, experimental systems and methods used in many studies. Here, indicate whether each material, system or method listed is relevant to your study. If you are not sure if a list item applies to your research, read the appropriate section before selecting a response.

Materials & experimental systems

n/a	Involved in the study	
[ ]	[x]	Antibodies
[ ]	[x]	Eukaryotic cell lines
[x]	[ ]	Palaeontology and archaeology
[ ]	[x]	Animals and other organisms
[x]	[ ]	Human research participants
[x]	[ ]	Clinical data
[x]	[ ]	Dual use research of concern

Methods

n/a	Involved in the study	
[x]	[ ]	ChIP-seq
[x]	[ ]	Flow cytometry
[x]	[ ]	MRI-based neuroimaging

Antibodies

Antibodies used

Primary antibodies
anti-Keratin 5, Biolegend, 905501, rabbit polyclonal, immunofluorescence, 1:15000
anti-Keratin 10, abcam, ab76318, rabbit monoclonal, immunofluorescence, 1:15000
anti-Ki67, abcam, ab15580, rabbit polyclonal, immunofluorescence, 1:15000
anti-beta-catenin, BD Biosciences, 610154, mouse monoclonal, clone 14, immunofluorescence, 1:5000
anti-CD49f (integrin alpha 6)-PE conjugated, ThermoFisher, 12-0495-82, rat monoclonal, clone NKI-GoH3, epidermal basal cell enrichment for single cell RNA analysis, 2 micrograms of antibody in 100 microliters of single cell suspension of skin cells (~106 cells)
anti-Digoxigenin POD (horseradish peroxidase-conjugated), Roche/Sigma Aldrich, 11207733910, sheep polyclonal, in situ hybridization, 1:5000
anti-digoxigenin AP (alkaline phosphatase-conjugated), Roche/Sigma Aldrich, 112093274910, sheep polyclonal, in situ hybridization, 1:5000
anti-GFP, abcam, ab290, rabbit polyclonal, immunoblotting, 1:1000

anti-Flag, Millipore Sigma, F1804, mouse monoclonal, M2 clone, immunoblotting, 1:1000

Secondary antibodies
goat anti-rabbit IgG - Alexa 488 conjugated Jackson ImmunoResearch, 111-545-144, immunofluorescence, 1:400
goat anti-rabbit IgG – biotin-SP conjugated, Jackson ImmunoResearch, 111-065-144, immunofluorescence, 1:5000
goat anti-mouse IgG – biotin-SP conjugated, Jackson ImmunoResearch, 111-065-166, immunofluorescence, 1:1000
goat anti-rabbit IgG – horseradish peroxidase conjugated, Jackson ImmunoResearch, 111-035-144, immunoblotting, 1:5000
goat anti-mouse IgG - horseradish peroxidase conjugated, Jackson ImmunoResearch, 115-035-166, immunoblotting, 1:5000

Validation

anti-keratin 5 – antibody has been used for immunofluorescence staining of mouse skin (eg. PMID 18039792). The expression pattern observed in mouse skin is similar to the pattern observed in fetal cat skin.
anti-keratin 10 – antibody has been used for immunofluorescence staining of mouse skin (eg. https://www.abcam.com/cytokeratin-10-antibody-ep1607ihcy-cytoskeleton-marker-ab76318.html). The expression pattern observed in mouse skin is similar to the pattern observed in fetal cat skin.
anti-Ki67 – antibody has been used for immunofluorescence staining of mouse skin and knockout validated in mice (eg. https://www.abcam.com/ki67-antibody-ab15580.html). The expression pattern observed in mouse skin is similar to the pattern observed in fetal cat skin.
anti-beta-catenin – anti--catenin antibodies have been used for immunofluorescence staining of embryonic mouse epidermis and developing hair follicles (PMID 12015971). The expression pattern in embryonic mice and cats using this antibody parallels the staining pattern observed for other anti--catenin antibodies.
anti-CD49f – we performed immunofluorescence using the unconjugated anti-CD49f antibody. Staining patterns in fetal cat skin samples paralleled those observed in other mammals (https://www.thermofisher.com/antibody/primary/query/cd49f). In addition, quantitative RT-PCR on enriched and unenriched cell fractions for genes representing different skin cell populations (eg. basal epidermis – Keratin 5, suprabasal epidermis – Keratin 10, dermis – PDGFR) parallel the results from the single cell RNA experiments.
anti-Digoxigenin POD (horseradish peroxidase-conjugated) – antibody has been used for in situ hybridization to recognize digoxigenin labeled probes (https://www.sigmaaldrich.com/catalog/product/roche/11207733910)
anti-digoxigenin AP (alkaline phosphatase-conjugated) – antibody has been used for in situ hybridization to recognize digoxigenin labeled probes (https://www.sigmaaldrich.com/catalog/product/roche/11093274910)
anti-GFP – antibody has been used to probe blots for immunoblotting (https://www.abcam.com/gfp-antibody-ab290.html). Fluorescence microscopy was used to visualize GFP-positive cell to confirm expression.
Anti-flag – antibody has been used to probe blots for immunoblotting (https://www.sigmaaldrich.com/catalog/product/sigma/f1804)

Eukaryotic cell lines

Policy information about cell lines

Cell line source(s): HEK293 cells were purchased from ATCC (CRL-1573)

Authentication: No cell authentication was performed

Mycoplasma contamination: Cell lines were not tested for mycoplasma.

Commonly misidentified lines (See ICLAC register): No commonly misidentified cell lines were used in the study.

Animals and other organisms

Policy information about studies involving animals; ARRIVE guidelines recommended for reporting animal research

Laboratory animals: The study did not involve laboratory animals.

Wild animals: Otherwise discarded embryonic cat tissues were recovered incidentally from pregnant feral cats at spay-neuter clinics in California after the spaying surgery. Viable embryos are recovered without compromising the health of the feral dams. Genomic DNA was collected from buccal swabs at cat shows with permission of the owner or by mail submission from cat breeders.

All tissue samples represent Felis domesticus embryos between stage 15 and 18. Embryo sex was not determined as it does not influence and is independent of tabby color pattern.

Field-collected samples: No field-collected samples were used in the study.

Ethics oversight: Sample collection and processing was conducted in accordance with a protocol approved by the Stanford Administrative Panel on Laboratory Animal Care.

Note that full information on the approval of the study protocol must also be provided in the manuscript.
